# Before the Potty Party: Preparing Practitioners for Toilet Training

**DOI:** 10.1007/s40617-025-01105-5

**Published:** 2025-11-10

**Authors:** Nicole A. Hollins, Brandon C. Perez, Rebecca L. Kolb, Timothy R. Vollmer, Stephanie M. Peterson, Holly C. Perez

**Affiliations:** 1 School of Education, Virginia Commonwealth University, Richmond, VA 23230 USA; 2 Special & Early Education Department, Northern Illinois University, DeKalb, IL 60115 USA; 3 Department of Educational Psychology, University of Minnesota, Minneapolis, MN 55455 USA; 4 Department of Psychology, University of Florida, Gainesville, FL 32611 USA; 5 Department of Psychology, Western Michigan University, Kalamazoo, MI 49008 USA; 6 Diplomate of the American Board of Family Medicine, Lexington, KY USA

**Keywords:** Autism spectrum disorder, Developmental disabilities, Ethics, Incontinence, Toilet training

## Abstract

There are limited resources specific to preparing for and developing individualized, intensive toilet-training programs for individuals with developmental disabilities. This has resulted in practitioners omitting critical steps within the program-development phase and hastily including unnecessarily intrusive training components, leading to unfavorable training contexts and variable caregiver buy-in. The purpose of this article is to discuss ethical and practical considerations for developing intensive toilet training programs. We do not provide guidance on toilet-training protocols, as this has been extensively described elsewhere. Instead, we offer recommendations to bridge the existing gap in the literature for toilet training planning that is focused on medical considerations, risk assessment, choice to participate, data collection systems, and caregiver collaboration.

Continence is a challenging skill to master and can be an even greater challenge for those diagnosed with developmental disabilities such as autism spectrum disorder (ASD; Dalrymple & Ruble, 1992). Toilet training is often an intensive and comprehensive process that, for many, requires significant ecological and programmatic modifications like other intensive assessments and interventions in applied behavior analysis (Deochand et al., 2020).

Intensive toilet training programs (ITTPs) may include (a) increased fluids, (b) functional communication training, (c) urine alarms, (d) elimination contingencies, (e) dry checks, (f) video models, and (g) scheduled toilet sittings (Francis et al., 2017; Kroeger & Sorensen-Burnworth, 2009; LeBlanc et al., 2005; Saral & Ulke-Kurkcuoglu, 2020; Simon et al., 2022). Implementing ITTPs often requires incorporating parental input (e.g., caregivers, legally authorized representatives) as well as other professional disciplines (e.g., teachers, physicians, occupational therapists; Donnelly & Karsten, 2024). Throughout the development and facilitation process, practitioners are responsible for selecting effective treatment components (BACB, 2020) and empowering clients to act as their own agents to improve their quality of life (Peterson et al., 2021). Doing so may strengthen the therapeutic relationship, increase bidirectional compassionate communication, and, most importantly, positively impact client outcomes (Reid et al., 1999; Rohrer et al., 2021; Slim & Reuter-Yuill, 2021). As such, there is value in discussing how practitioners prepare to facilitate ITTPs that are individualized for each client’s unique needs, skills, and preferences.

Best-practice guidelines, decision-making tools, checklists, and supplementary resources assist practitioners in making ethical and data-based decisions. Such resources exist across many topic areas, including risk assessments for functional analyses (Deochand et al., 2020), treatment selection for interfering behaviors (Geiger et al., 2010), therapeutic service modality (Rodriguez, 2020), measurement procedures for interfering behaviors (LeBlanc et al., 2016), compassionate collaborative skills (Rohrer et al., 2021), and appropriateness of preference assessments (Virués-Ortega et al., 2014). However, only a few such resources are available to prepare ITTPs for individuals with developmental disabilities.

Frank et al. (2020) developed a decision-making protocol to inform individualized modifications to both rapid and interval toilet training interventions. Frank et al. found the decision-making protocol to be helpful, as there was no one-size-fits-all protocol for ITTPs. Similarly, Cagliani (2022) created an evidence-based guide for toilet training in early childhood classrooms that described procedures and considerations for this setting. Although Cagliani et al. and Frank et al. provided guidance on procedural implementation and decision-making while implementing ITTPs, they did not discuss preparatory steps for initiating ITTPs, including medical considerations, assessment of risk, choice to participate, or data collection systems. These considerations are essential due to the vulnerable nature of toilet training, which necessitates prioritization of client self-determination, autonomy, and safety.

The purpose of this article is to discuss ethical and practical considerations when preparing to facilitate ITTPs. This article is not intended to synthesize the toilet training literature or individual procedures (see reviews by Francis et al., 2017; Kroeger & Sorensen-Burnworth, 2009; Saral & Ulke-Kurkcuoglu, 2020; Simon et al., 2022). Rather, this article provides practitioners with practical guidance for planning ITTPs that are aligned with evidence-based best practices focused on medical considerations, risk assessment, choice to participate, data collection systems, and caregiver collaboration.

## Medical Considerations

Although bowel and bladder incontinence can be expected in a client who has never been toilet trained, each can also be secondary to an underlying medical condition or impacted by medication use. Therefore, behavior analysts implementing ITTPs should familiarize themselves with a client’s medical history and recognize indicators that strengthen the need for collaboration with the client’s medical providers (Bacotti et al., 2023). This is important both for optimizing client skill acquisition and for adhering to the BACB Ethics Code (i.e., considering medical needs [2.12] and consulting with other providers [3.06]; BACB, 2020). This section was written in partnership with a physician (sixth author) to highlight the significant benefit clients, caregivers, behavior analysts, and medical providers alike stand to gain through collaboration.

Acknowledging medical considerations may positively impact client comfort and safety during ITTPs. While it is not within the scope of a behavior analyst to assign medical diagnoses or adjust client medication dosages (BACB, 2020), it is within their scope to recognize variable trends in client data based on medication dosage adjustment, which may suggest the need for medical evaluation.

There are several symptoms that should be monitored before implementing ITTPs and throughout the training process. For example, a client with a neurological condition may lack the neuromuscular framework required for bladder or bowel continence. In this case, enrolling them in ITTPs could be contraindicated and unethical. Preemptive medical collaboration could spare the client an unnecessary, ineffective treatment.

During ITTPs, clients are offered fluids to increase the motivating properties of a full bladder, which, in turn, increases the probability of elimination (Simon et al., 2022). A client with a history of congenital heart or kidney disease may have a daily fluid restriction in place, which could impact safe fluid parameters. Additionally, high blood sugar in a client with diabetes can cause the symptom of polyuria (i.e., large volume urine output), requiring more frequently scheduled sit opportunities or adjustment of their diabetes medications prior to or during the ITTP, particularly if their highest preferred food items contain high sugar content. Any client demonstrating a sudden, seemingly unexplained change in elimination patterns from a previous baseline (e.g., new distress while eliminating, blood in urine or stool, diarrhea) should prompt the practitioner to consider pausing the ITTP until the client can be evaluated for acute infection or illness, especially when occurring alongside other commonly recognized signs of infection (e.g., fever, vomiting, decreased appetite; American Academy of Family Physicians [AAFP], 2011). This promotes client safety and accounts for how acute infections can temporarily and adversely impact continence patterns unrelated to the client’s true potential for continence and ITTP success.

For clients with an extensive history of stool refusal, it is imperative to seek the input of a physician (Friman et al., 2006, 2020) to support not only the client’s successful acquisition of continence, but to record and understand trends in stool output accurately. A client with long-standing constipation or stool refusal who suddenly develops loose stool leakage, for example, may result in misidentifying interventions to loosen stools as successful, when actually, this can be a complication of prolonged constipation (i.e., fecal overflow incontinence; Columbo et al., 2015).

Many commonly used medications (e.g., antihistamines, antipsychotics) have constipation or urinary retention as a side effect (Niemczyk et al., 2018). These side effects can decrease the number of daily voids or cause painful straining during attempts to void, which may reduce opportunities for the client to contact reinforcement and introduce an aversive stimulus (i.e., pain during elimination) before accessing reinforcers (e.g., pressure relief and preferred items; Bacotti et al., 2023). Collaboration with medical prescribers can determine if medication adjustment or specific therapies (e.g., laxatives) may assist the client in gaining continence (Axelrod et al., 2016; Call et al., 2017; Niemczyk et al., 2018; Vasconcelos et al., 2006).

Behavior analysts have unique expertise in recording observable behaviors (e.g., the frequency of urine eliminations) to determine whether there are anomalies or stable trends (Cooper et al., 2020). Doing so allows behavior analysts to objectively inform both medical providers and caregivers on how new or discontinued interventions impact voiding behavior. Rudimentary knowledge of toileting medical considerations, interdisciplinary collaboration with caregivers and physicians, proper elimination posture, and appropriate fluids are all medical considerations that should be evaluated when developing ITTPs. Although not exhaustive, these examples highlight how significantly a client’s medical history may impact their voiding habits and potential for successful ITTP completion and why it is prudent to collaborate with medical providers before and during ITTPs.

## Risk Assessment

When preparing to facilitate ITTP, it is recommended that practitioners evaluate all potential risks for all clients, particularly for clients who were previously nonresponsive to ITTPs or who had aversive experiences with toileting in the past. A risk assessment entails a structured clinical decision-making process that involves the organization of ethical and safety considerations (Wiskirchen et al., 2017). Although there is a dearth of toileting risk–benefit assessments, there is immense value in discussing how to assess risk levels before proceeding with toileting programs. Wiskirchen et al. (2017) categorized risk assessment into four domains: clinical experience, behavior intensity, environment, and staff support. This section will discuss risk assessment recommendations for ITTPs under these domains.

### Clinical Experience

Approximately 47,000 behavior analysts were credentialed between 2017 and 2024 and, thus, were categorized as early career professionals (BACB, 2022). It is likely, then, that a similar proportion of behavior analysts who facilitate ITTPs are also early career professionals. As such, the extent of clinical experience likely influences the effective development and implementation of ITTPs. Behavior analysts should evaluate their level of engagement, comfort, and scope of competence (Brodhead, 2018) across their past ITTP experiences. Like other comprehensive assessments and interventions, the implementation of ITTPs includes multiple levels of engagement (i.e., observe, implement, train, develop, and program for generalization and maintenance across settings). Thus, if a practitioner has primarily participated in the direct implementation of ITTPs for several clients but has had limited to no opportunities to develop or train others to implement ITTPs, it is recommended they seek out additional supervision or mentorship prior to conducting an ITTP independently to mitigate any risk of harm (Sellers et al., 2016).

### Behavior Intensity

Interfering behaviors (e.g., emotional responding, destructive behavior, disruptive behavior, aggression toward others, property destruction, self-injurious behavior, or toileting refusal) may present throughout the training context*.* It is essential to identify client risks based on the current magnitude or topography of the interfering behaviors and to develop procedural adaptations to minimize risk during ITTPs (Doan & Toussaint, 2016). For example, Doan and Toussaint measured the frequency of interfering behaviors throughout baseline and toilet training. One participant’s interfering behavior increased as scheduled sits were introduced. To ensure client and caregiver comfort, the sitting schedule was reduced from 5 to 3 min, which resulted in decreased interfering behaviors throughout the intervention. Perez et al. (2021) also measured interfering behavior that occurred during toilet training and found that it generally decreased throughout the course of the intervention for most participants. Additionally, interfering behavior was more commonly observed during trials in which the participant had a urinary or bowel movement accident. These examples highlight the importance of recording and modifying training components to ensure ethical practices are upheld when interfering behavior occurs.

Furthermore, if a client engages in interfering behavior and a termination criterion is already established within their daily programming, it is recommended to update the criterion based on the idiosyncrasies of ITTPs. That is, a client may have a termination criterion of five or more instances of self-injurious behavior (e.g., hand-to-head hitting) during regular daily programming. Within the ITTP, the termination criterion may decrease from five instances to two instances to ensure client and staff safety in the bathroom. Alternatively, there may be other scenarios where clients do not typically engage in a high magnitude of interfering behavior in their daily programming. In that case, the practitioner should facilitate a proxy simulation of interfering behaviors while using behavioral skills training (Parsons et al., 2012) to support staff in maintaining client safety, in the case that interfering behaviors are arbitrarily displayed throughout the ITTPs.

### Environment

It is recommended to evaluate the risk of the physical environment where toilet training will occur to enhance comfort and ensure privacy as a preparatory step. Given the substantial differences between training environments (e.g., clinical settings, school settings, and home settings), it is important that all training components be individualized to the training environment. Furthermore, collaborating with other professionals (Slim & Reuter-Yuill, 2021) at the onset of ITTPs may support identifying environmental features or accommodations that will impact client comfort.

Environmental safety precautions during ITTPs may include reserving a single-person bathroom, using dividers to maintain privacy (Wohofsky et al., 2023), removing hazards from the environment (Matson, 2012), and padding the sharp corners (Wiskirchen et al., 2017). Additional environmental accommodations may also include getting the client accustomed to bathroom sounds (e.g., toilet flushing or hand dryer; Bacotti et al., 2023), neutral toilet seat temperature, or selecting bathrooms that have child-size toilets that allow feet to remain on a flat surface (Friman et al., 2006). To prepare for an accident, it is recommended to protect the furniture (e.g., liquid-resistant rug or cloth) and ensure that additional clothing is available. Preparing the environment to accommodate these variables based on individual preferences contributes to an individualized training approach and minimizes potential inadvertent exposure to aversive stimuli.

### Support Staff

Confirming staff availability is an important step when preparing to facilitate ITTPs. As previously mentioned, ITTPs are typically fast-paced and involve support staff (i.e., caregivers or behavior technicians). Prior to facilitating ITTPs, it is essential that roles and responsibilities be clearly outlined for support staff (e.g., Who is directly working with the client? Who is collecting data? Who is cleaning up the area?). It is also important that staff be trained to fluency (as measured by meeting predetermined mastery criteria on procedural fidelity checks) on ITTP components as well as behavior management procedures (Poling et al., 2012). This should include staff instructions, modeling, rehearsal, and feedback (Parsons et al., 2012) specifically for how to respond to successful eliminations and accidents, how to maintain client privacy when transitioning to the bathroom, and how to communicate to other members in the environment that either an accident has occurred (i.e., clean up support is needed) or a bathroom is occupied. Then, procedural fidelity should be measured to ensure all components of the ITTP are implemented correctly (Perez et al., 2020). Furthermore, practitioners should explicitly communicate the necessary time requirements for ITTPs directly to support staff as risk increases for the clients if insufficient support staff are available (Wiskirchen et al., 2017).

## Choice to Participate

### Toileting Readiness Skills

Toileting readiness skills are usually based on longitudinal studies including typically developing children (Schum et al., 2002). In general, some researchers have identified readiness skills for toileting to include a client being able to walk, follow one-to-two step instructions, hold bladder for at least 2 h, remove clothing, and demonstrate dissatisfaction with a soiled diaper (Baird et al., 2019). Other researchers have recommended clients be of a certain developmental age (Schum et al., 2002) or have mastered specific readiness skills (Kaerts et al., 2012) before attempting ITTPs. Furthermore, other practitioners have implemented a child-oriented, intrinsic approach (Brazelton et al., 1999) or a parent-oriented, structured approach (Azrin & Foxx, 1974) to promote continence. Nonetheless, the literature on toilet training readiness is unclear and divided (Kaerts et al., 2012). To date, there is *no* consensus on readiness skills that are required, or even necessary, before beginning ITTP.

The variability in the literature suggests that readiness skills may be informative for planning ITTPs, but they are not essential for toilet training success (Cagliani, 2022). For example, Johnson et al. (2021) conducted a systematic literature review focused on dosage and procedural modifications for ITTPs for individuals with developmental disabilities. Of the 55 studies evaluated, only a small percentage of participants were able to engage in toilet readiness skills, yet many of the ITTPs were successful. Thus, toilet training should *not be* delayed solely based on a client demonstrating readiness skills.

As toilet training is an important developmental milestone, one may anticipate questions from caregivers related to readiness. In these scenarios, behavior analysts should validate their concerns, answer questions, share the short- and long-term impacts of gaining continence, and ultimately allow data to inform decisions about beginning toilet training. It is important to note that the caregiver and the client have the ultimate choice to consent or assent to ITTPs, regardless of their current skill set.

### Consent

Receiving parental consent is a critical first step, and the process may vary depending on the setting. A client may be referred to participate in ITTPs by parents (Foxx & Azrin et al., 1973; Hanney et al., 2012; Henriksen & Peterson, 2013; McLay et al., 2015; Mruzek et al., 2016; Perez et al., 2020; Post & Kirkpatrick, 2004; Taylor et al., 1994) or an interdisciplinary team (Call et al., 2017; Greer et al., 2016; LeBlanc et al., 2005; Radstaake et al., 2013; Sells-Love et al., 2002). When seeking parental consent, the primary consideration for practitioners should include how ITTP information is initially presented to parents. Van Aggelpoel et al. (2018) facilitated six focus group discussions to identify how parents prefer to receive information related to ITTPs, how to best inform them, and who are trustworthy advocates. The results of this study indicated that parents preferred to receive toilet training information in a brochure format including colorful infographics, understandable language, and limited scientific terminology. Thus, practitioners are encouraged to develop a setting-specific brochure (or one-pager) that outlines the toileting process, time commitment, and frequently asked questions as an informational resource for caregivers. This may increase caregiver active engagement during the consent meeting, increase comprehension of the training process, and assist caregivers in posing detailed questions specific to their child’s individual needs.

### Assent

Although gaining independent toileting skills has significant short- and long-term benefits for the individual being trained (CDC, 2020) and caregivers may initially provide consent for their child to participate, it is also important to gain assent from those who are receiving services. Doing so aligns with personal choice to participate (BACB, 2020) and self-determination (Peterson et al., 2021). However, there is limited-to-no literature focused on gaining client assent as a preparatory step to facilitating ITTPs. Thus, this section will discuss the nuances of assessing and recording assent during ITTP.

Assent is defined as an individual “who is not legally authorized to provide their own consent for participation affirmatively confirming their willingness to participate” (Morris et al., 2021, p.1300). Morris et al. provided several example formats for gaining assent from clients who can express assent both directly and indirectly, including using verbal statements (e.g., asking the client if they would like to participate), observing assent behaviors (e.g., walking into the bathroom or positive affect), using a psychomotor response (e.g., placing a Velcro happy or sad smiley face on bathroom visual), or providing a tutorial (e.g., requesting the client to complete a segment of the procedures). As with obtaining consent, a necessary part of the assent process, whenever possible, is to ensure procedures are explained to the client in an age and developmentally appropriate manner (e.g., social story, visual depiction of procedures). Additionally, it is important to assess client understanding of the ITTP, including aspects such as its benefits, time commitment, and toileting components. It is essential to not only assess assent initially but to monitor assent behaviors throughout the ITTP.

As both consent and assent are typically obtained at the onset of training, it is possible that some clients may intermittently dissent or opt out of specific toileting components or the entire toileting program. These cases are particularly challenging to navigate. In parallel, there are many scenarios in which a client may refuse treatment, but the caregiver may prefer to continue with the medically necessary treatment. For example, a child may protest going to the dentist to get a rotten tooth extracted, but the caregiver may prefer to proceed with the tooth extraction as it is in the best interest of the client. In fact, many imaginable circumstances exist when the absence of child assent would not (or should not) disrupt a necessary procedure or routine (e.g., bathing, getting stitches, brushing teeth, and so on). Similarly, in toilet training contexts, a parent may be comfortable or even personally compelled to proceed with a toileting component (e.g., scheduled toilet sits) even if the client verbally protests while sitting on the toilet (Wilczynski et al., 2015). These cases must be explicitly discussed with the treatment team as it is essential for behavior analysts to engage in trauma-informed and compassionate service delivery while establishing a physically and emotionally safe environment where the client’s needs are being met and simultaneously balancing longer-term health and social outcomes (Guarino et al., 2009; Penney et al., 2023; Rajaraman et al., 2022).

One solution may be to pair the bathroom with reinforcement while slowly introducing the client to the bathroom stimuli and the toileting components to gain client assent, which may ultimately increase client preferences toward the toileting program. If during this pairing process, the client assents to certain toileting components (e.g., scheduled sits) but not to others (e.g., accepting fluids), behavior analysts should explicitly record these instances to support modifying the ITTPs. It is essential to emphasize that assessing assent should be an ongoing process to increase the probability of client comfort with the toileting procedures. This may include teaching the client to communicate when they have to use the bathroom (need a break or want to leave the restroom), asking ongoing verbal questions to clients before each scheduled sit (e.g., “Are you excited about the next potty party?”), and by closely monitoring assent behaviors (e.g., observing a smile or positive affect while transitioning to the bathroom; Morris et al., 2021). It is also recommended that practitioners discuss with caregivers, both proactively and throughout training, that interfering behaviors may present during toilet training and emphasize that training will stop at any point that the client protests beyond a predetermined criterion. It is equally critical to discuss with caregivers the importance of the client being comfortable throughout ITTPs, and the behavior analysts may need to modify or omit a toileting component at any point during the training context for the best interest of the client (Ethics Code 3.01; BACB, 2020). Nonetheless, an important caveat must be stated. If all assent adaptations have been exhausted and the client continues to engage in behaviors that suggest dissent, then it is recommended that practitioners re-evaluate the risk–benefits for the ITTPs.

## Data Collection Systems

The next step in preparing for ITTP includes establishing robust data collection systems to record toileting behaviors during baseline and intervention. Collecting baseline data will aid the practitioner in evaluating client performance in the absence of an independent variable, provide guidance in setting initial criteria for reinforcement, and maintain the merits of objective measurements versus subjective opinion (Cooper et al., 2020). Additionally, consistent data collection systems throughout the training may support considering medical needs (2.12; BACB, 2020), designing assessments and treatments (2.13; 2.14; BACB, 2020), and continuously evaluating progress (2.18; BACB, 2020). This section will highlight essential components that should be included in data collection systems for ITTPs.

### Enuresis and Encopresis

Both urine and bowel movement eliminations should be closely monitored throughout baseline and training sessions. A primary goal for ITTPs includes increasing the proportion of in-toilet eliminations and decreasing accidents (Francis et al., 2017). It is recommended to use non-frequency-based data analysis, such as percentage, to measure eliminations and evaluate the proportion of scheduled sits with in-toilet eliminations rather than using frequency or rate, as these vary across days (Cagliani et al., 2021; Cooper et al., 2020; Doan & Toussaint, 2016; Greer et al., 2016; LeBlanc et al., 2005; McLay et al., 2015). For example, if a client eliminated in the toilet seven out of nine scheduled sits, the accuracy of in-toilet eliminations would be calculated by dividing the frequency of in-toilet eliminations by the total opportunities (i.e., in-toilet elimination plus accidents) and multiplying that number by 100 (7 / 9 = 0.78 × 100 = 78%). By selecting percentage as the primary method, practitioners are simultaneously collecting frequency, which may support additional analyses (i.e., scatter plots). Evaluating the data in this format may aid in further analyzing the primary dependent variables.

### Communication Training

A critical component of ITTPs includes prompting a communicative response before each scheduled bathroom visit (Francis et al., 2017; LeBlanc et al., 2005). To adequately prepare for ITTPs, practitioners should establish a data collection system that records self-initiations (e.g., gestures, signs, picture exchange communication systems, speech-generating devices, or vocal speech; Greer & Ross, 2004; Skinner, 1957) when transitioning to the bathroom. If the client does not often transition to the bathroom due to wearing diapers or other physical abilities, it is recommended to transition from changing diapers in other settings (e.g., treatment rooms) to the bathroom to aid in the transfer of stimulus control for in-toilet eliminations. Nonetheless, developing a data collection system that records self-initiations will support the practitioner in developing an individualized communication procedure to embed within the ITTP based on the client’s current repertoire.

Researchers have used various data collection methods to record self-initiations during ITTPs (Francis et al., 2017; Simon et al., 2022). Self-initiation data collection may be challenging in that a client may self-initiate in the absence of a scheduled sit, or a client may self-initiate in the absence of eliminating in the toilet. Thus, using data collection methods, such as frequency or rate (Greer et al., 2016; Perez et al., 2020), may support recording the total number of responses over the training days. However, researchers have also used percentage as a data collection method when recording communication throughout the ITTPs (Doan & Toussaint, 2016; LeBlanc et al., 2005; Perez et al., 2021). Collecting data on self-initiations allows practitioners to analyze if the client will require a toileting schedule long-term or if they will be able to request the bathroom in the absence of a schedule independently. Regardless of the data collection type, it is recommended to use continuous measurement (Kostewicz et al., 2016) when recording self-initiations throughout ITTPs.

### Interfering Behaviors

Several components within ITTPs may evoke interfering behaviors, including when the client transitions to the bathroom, during the scheduled sit, during a trial in which a toileting accident has occurred, the presence of the urine alarm, or after a scheduled sit where no in-toilet eliminations occurred (i.e., reinforcement was not provided). However, recording every topography or magnitude of interfering behaviors may not be feasible (Najdowski et al., 2009) and not every measurement procedure is appropriate for every type of interfering behavior (LeBlanc et al., 2016). Thus, it is essential to collect the type of data needed to make timely and meaningful data-based decisions about the ITTPs (Bushell & Baer, 1994).

LeBlanc and colleagues (2016) provide a useful decision-making framework to support practitioners in selecting measurement procedures for interfering behaviors. Seven different measurement procedures are discussed, including event recording, duration recording, latency recording, intensity recording, permanent product recording, partial-interval recording, and momentary time sampling. Historically, published ITTPs have not included objective data on interfering behaviors or the nuances surrounding accidents within the training context beyond stating the client produced emotional reactions and responses (Ando, 1977; Butler, 1976) or appeared to be highly agitated and distressed (Doan & Toussaint, 2016; Flood & Luiselli, 2016). While recently, a small proportion of researchers have included interfering behaviors in their data collection systems (Perez et al., 2021), a description of such systems does not appear to be a standard component for ITTPs. It is recommended to use evidence-based decision-making frameworks (LeBlanc et al., 2016) and clinical judgment to determine a data collection method that would be appropriate, accurate, and feasible given client characteristics and the training settings.

For example, Louis is a five-year-old student in an early childhood special education classroom with 15 other students. The teacher and paraprofessional are hesitant to collect data for the consulting behavior analyst due to other classroom task demands. Louis reliably displays interfering behaviors (i.e., crying) when his diaper is wet, which disrupts classroom activities and may indicate discomfort. Using the clinical decision model by LeBlanc and colleagues (2016), the behavior analyst selected partial interval recording to measure the presence of crying during specific time intervals during the school day. As classroom staff are hesitant to collect data, developing a data collection system with a low response effort is essential. To this end, the behavior analyst develops a simple data collection system that can be laminated and placed next to the sink in the bathroom. Furthermore, staff would only be required to move a Velcro® icon for a specific interval if crying is observed during that time. This system ensures that data is collected consistently without interrupting other classroom responsibilities. It is imperative that data collection systems are individualized to the setting and capture essential data to inform procedural modifications. As interfering behaviors communicate discomfort or dissent, it is critical that behavior analysts closely monitor assent throughout each toileting component. The following section will discuss considerations and recommendations for doing so.

### Assent Behaviors

In our field, monitoring assent and collecting data on assent behaviors is relatively new. Recently, researchers have developed data collection systems to measure indices of happiness within a functional analysis context using partial interval recording (Thomas et al., 2019). Within the context of feeding programs, researchers have also watched recorded feeding sessions and used a Likert scale to measure perceived indices of happiness or unhappiness (Phipps et al., 2022). Although indices of happiness do not reflect all components of assent, it is a proxy measure that may support the individualization of a toileting program.

Given that little-to-no toileting literature explicitly measures assent, it is recommended to appropriately select a continuous data collection method that accurately captures assent behaviors throughout the training, as doing so may inform procedural modifications and may impact overall social validity. To do so, a behavior analyst should determine the topography by which assent will be evaluated (i.e., using verbal statements, observing assent behaviors, using a psychomotor response, or providing a tutorial), define assent individualized to the client, and identify an appropriate continuous data collection method. For example, a behavior analyst may determine that assent will be monitored by observing behaviors indicative of assent throughout the training. Once the discriminative stimulus has been placed (e.g., “Let’s go potty), the behavior analyst may use 30-s partial interval recording to measure client assent (e.g., walking to the bathroom, sitting on the toilet) and dissent (e.g., refusal or avoidance) throughout the ITTPs. The total percentage of intervals with assent should be closely monitored throughout the training to monitor safety and maintain client comfort.

## Caregiver Collaboration

Behavior analysts are ethically obligated to involve caregivers throughout service provision (BACB, 2020). Moreover, research notes that caregiver involvement supports skill acquisition, generalization, and maintenance for children with developmental disabilities (Althoff et al., 2019). Thus, this section will discuss considerations for practitioners seeking caregiver feedback before facilitating ITTPs.

### Preferences

Caregiver and client preferences should be assessed throughout every component of ITTPs (Cagliani, 2022). Practitioners may conduct interviews (Simmons et al., 2024) or provide caregivers with questionnaires to gather preliminary information about the child’s previous toileting history and preferences before beginning training (Dalrymple & Ruble, 1992; Matson et al., 2010; Maskey et al., 2012; Strauss & Zigman, 1996; Von Gontard et al., 2010). Some questions may focus on their child’s preferred elimination positions (e.g., standing or sitting), preferred items or activities (Tullis et al., 2011), and preferred underwear. Other questions may be focused to the caregivers preferred method of communication (e.g., phone calls, text messages, or e-mails) for virtual support outside of clinical hours, comfort with their child using hand sanitizer between scheduled sits, and ensure training components are culturally appropriate (e.g., elimination position or training materials reflective of the child’s ethnicity). As a preliminary step, gathering preference feedback from the child and caregiver will directly support practitioners in developing an individualized training procedure.

In ITTP, active caregiver involvement is highly valuable, as their role is essential for sustaining toileting procedures beyond the training date in various contexts (Frank et al., 2020; Simmons et al., 2024). However, the extent to which caregivers are willing to participate may differ. Therefore, discussing their preferred level of engagement from the outset is advisable. For example, caregivers may prefer to only implement specific components of the ITTP (e.g., providing reinforcement after in-toilet eliminations). In this scenario, practitioners may determine that further training is needed for toileting components that caregivers did not directly implement. Practitioners may simultaneously seek feedback from caregivers to assess their confidence in implementing the procedures independently at home. If caregivers are unable to attend training sessions due to scheduling conflicts, it is recommended that practitioners coach caregivers on implementing the toileting components with adequate fidelity. This approach will help facilitate the generalization and maintenance of in-toilet eliminations across multiple settings.

### ITTP Proposal

Another critical step in preparing for ITTP involves presenting the proposed treatment components to the caregiver, accompanied by baseline data. Engaging caregivers in the decision-making process before beginning the ITTP may contribute to identifying variables that affect parent–child interactions, identifying any relevant contextual variables (e.g., cultural variables, aversion to the bathroom, hard time with the flush sound, etc.), increasing the efficacy of generalization programming (Becraft et al., 2024), and assessing comfort (Saral & Ulke-Kurkcuohlu, 2020). Furthermore, collaborative involvement may increase the external validity of the procedures. Such sensitivity represents the equivalent of “good bedside manner” and may strengthen the overall collaborative partnership and support the development of ITTPs specific to the families’ wants and needs throughout each phase of the toileting program. When presenting toileting components informed by baseline data to caregivers, it is recommended to do so using the previously discussed practices (see “Choice to Participate”).

### Data Collection

Once the caregiver and practitioner come to an agreement on the proposed toileting components, it is important to discuss the feasibility and needed modifications to the data collection system (e.g., an abbreviated version or electronic) to support caregivers in collecting data in the home setting. While data collected in the home setting may not be used as the primary variable to determine procedural changes, it is valuable for caregivers to report macro changes to practitioners (i.e., treatment effects or outcomes; Morris et al., 2022). This is also applicable to the treatment team. That is, to increase the probability of in-toilet eliminations across multiple settings, it is recommended to inform the treatment team (e.g., teacher, caregivers, speech-language pathologist, etc.) who may often interact with the child on the agreed upon toileting procedures and seek input on data collection feasibility (Becraft et al., 2024). Doing so as a preliminary step may help treatment team members understand the scope and process for ITTP.

## Conclusion

Although the literature on toilet training is robust, our field currently lacks a practical set of recommendations addressing the essential preparatory steps that should be completed before facilitating ITTPs. A practical set of recommendations is valuable to discuss as doing so may support practitioners in adequately preparing for and developing ITTPs. Furthermore, an individualized or person-centered approach to toileting programs may encourage client self-determination across toileting skills. By prioritizing client safety and dignity, we have offered recommendations to bridge the existing gap in the literature for ITTPs focused on essential preparatory steps: medical considerations, risk assessment, choice to participate, data collection systems, and caregiver collaboration.

The benefits of gaining continence have been well researched (Francis et al., 2017). Yet, the omission of essential steps within the program development phase and the hasty inclusion of intrusive interventions may yield an unpleasant training context for the clients and implementers. As the field continues to make essential steps toward compassion and individualized treatments, behavior analysts must consider an amalgamation of factors with toilet training that centers compassion (Rodriguez et al., 2023) and promotes client comfort (Hanley, 2021). We encourage more scientist–practitioners to continue to expand the research in this area, particularly toward highlighting toilet training idiosyncrasies and methods to assess assent throughout toileting programs for individuals with and without developmental disabilities. Future research may also address practical recommendations and considerations regarding post-toilet training. It is our hope that the discussion will be meaningful for practitioners who facilitate ITTPs and inspire future practical research in this area.

## Data Availability

Data sharing is not applicable to this article as no data sets were generated or analyzed during the current manuscript.
